# HLA class I restricted epitopes prediction of common tumor antigens in white and East Asian populations: Implication on antigen selection for cancer vaccine design

**DOI:** 10.1371/journal.pone.0229327

**Published:** 2020-02-27

**Authors:** Wei Hu, Meifang He, Liangping Li

**Affiliations:** 1 Clinical Trials Unit, The First Affiliated Hospital of Sun Yat-sen University, Sun Yat-sen University, Guangzhou, China; 2 Institute of Clinical Oncology, The First Affiliated Hospital of Jinan University, Jinan University, Guangzhou, China; 3 Laboratory of General Surgery, The First Affiliated Hospital of Sun Yat-sen University, Sun Yat-sen University, Guangzhou, China; Duke University School of Medicine, UNITED STATES

## Abstract

Tumor antigens processed and presented by human leukocyte antigen (HLA) Class I alleles are important targets in tumor immunotherapies. Clinical trials showed that presence of CD8+ T cells specific to tumor associated antigens (TAAs) and tumor neoantigens is one of the main factors resulting in tumor regression. Affinity prediction of tumor antigen epitopes to HLA is an important reference index for peptide selection, which is highly individualized. In this study, we selected 6 CTAs (cancer-testis antigens) commonly used in cancer immunotherapy and top 95 hot mutations from the Cancer Genome Atlas for analyzing potential epitopes with high affinities to the common HLA class I molecules in white and East Asian population, respectively. The results showed that the overall difference in CTAs epitope prediction is small between the two populations. Meanwhile, there is a linear relationship between the CTAs peptide length and the relative overall epitope occurrence. However, the difference is bigger for epitopes prediction of missense mutations between the two populations. It is worth noting that, both in the two populations, the single point mutations with the highest incidences have the lowest epitope occurrences while the mutations with the highest epitope occurrences are with low mutation incidences. This may be the result of long-term selection by the host immunosurveillance. Frameshift/inframe indel mutation neoantigens are between CTAs and spot mutation neoantigens in the relationship between peptide length and predicted epitope number. Our results help provide clues for tumor antigen and epitope selection in cancer vaccine design.

## Introduction

T cells play a leading role in immunity to cancer in the majority of successful cancer immunotherapies, including immune checkpoint inhibitor, adoptive T cell transfer and tumor vaccine [1–4]. CD8+ T cell is one of main effectors resulting in tumor regression. Immune monitoring indicated that CD8+ T cell response targeting tumor antigens is an important index for immunotherapeutic effect.

T cell-mediated clinical trials need to predict the epitopes of tumor antigens with high HLA binding affinity *in silico*, for selecting peptide for cancer vaccination therapy or immune-monitoring of tumor specific T cells during the trial. The candidates in form of synthesized peptides or peptides expressing mRNAs were used to stimulate specific CD8+ T cells *in vitro* for T cell adoptive therapy or *in vivo* for tumor vaccine therapy [3, 5, 6]. Tumor antigens mainly classify into tumor associated antigens (TAAs) and tumor specific antigens (TSAs). TSAs comprise mutated neoantigens [7]. For safety and immunogenicity considerations, one kind of commonly used TAAs are cancer-testis antigens (CTAs) which are expressed exclusively in germ cells of the testis and fetal ovary but are silent in normal somatic cells [8–10].

The specific activation of CD8+ T cells is based on the antigenic epitope recognition by T cell receptor, which is restricted by human leukocyte antigen (HLA) [11–13]. All individuals express six HLA class I alleles and a population in an area has its own HLA allele frequency distribution. The affinity of a peptide to HLA determines whether the peptide can be presented. In this study, we selected 6 CTAs commonly used in cancer immunotherapy and 95 top hot mutations from the Cancer Genome Atlas to predict potential epitopes which may be presented by the common HLA class I molecules in white and East Asian population, respectively.

## Materials and methods

### HLA class I allele frequency ranking analysis

We collected HLA class I allele data from the Allele Frequency Net Database (AFND). The data collection criteria were as follows: 1). Alleles start from HLA-A*01:01 to HLA-C*18:03. 2). Choose two Ethnic Origins: Caucasoid or Oriental for white and East Asian population, respectively. 3). Select allele Level of resolution > = 4. Combine data from different populations, arrange the data in allele frequency from large to small and calculate the rate of HLA allele positive population using *Hardy-Weinberg equilibrium* [14], with the equation *p* = 1-(1-*q*)^2^, where *p* is the rate of positive population, *q* is the HLA allele frequency.

### Selection of CTAs

We selected 6 CTAs including KK-LC-1, MAGE-A1, MAGE-A4, NY-ESO-1, PRAME, SSX2, which were commonly used in tumor immunotherapy with high security record in clinical research, for Class I epitope prediction [15, 16].

### Selection of top somatic mutations from TCGA

We collected the top 100 somatic mutations by incidence with amino acid sequence change, including missense variant, frameshift variant, inframe deletion and inframe insertion, from all kinds of cancers in TCGA; arranged the data and removed three stop codon gain mutations (chr9:g.21971121G>A, chr1:g.200857892delA, chr6:g.30653271delT), B2M mutation (chr15:g.44711583delCT), PAX2 3’ Prime UTR mutation (chr10:g.100827567delC), and finally selected the rest 95 mutations for the following analysis (S1 Table).

### Tumor epitopes prediction

We chose whole protein sequences of CTAs and mutated peptide sequences to predict potential epitopes binding to HLA class I molecules. Mutated amino acids of spot mutations were flanked on both sides by 10 additional normal amino acids while the frameshift mutations were flanked by 10 additional normal amino acids on upstream and the rest mutated sequence on downstream. NetMHCpan4.0 algorithm was selected for HLA class I molecule restricted epitope prediction. The epitope length was restricted to 8–11 mer. Set rank threshold for Strong Binding (SB) < = 0.5 and Weak Binding (WB) < = 2.0 according to the default values.

## Results

**Fig 1:**
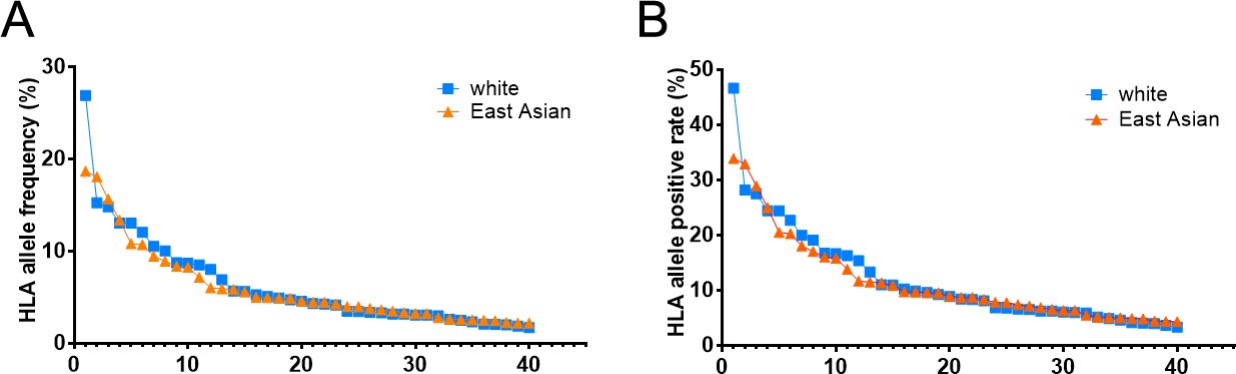
HLA allele frequency and positive rate in white and East Asian populations. The top 40 HLA alleles in white and East Asian populations were selected and displayed.

To get HLA class I allele frequency of white and East Asian populations, total number of 1442312 white individuals from 97 population samples and 322437 East Asian individuals from 87 population samples were collected respectively. HLA allele information was rearranged according to relative criteria (Method). The distribution and HLA allele frequency are different in white and East Asian populations. In white population, the top commonest HLA-A*02:01 accounting for 26.9% is far more than the second commonest HLA-A*01:01 (15.2%), while the trend changes more slowly in East Asian population (Fig 1). We selected the HLA alleles with positive rate > 0.001% from each population for the following epitope analysis (S2 Table).

Due to the accuracy of the NetMHC prediction based on artificial neural networks, we selected only strong binding results (%Rank < = 0.5) for the next analysis. For each population we first calculated the SB epitope numbers of each HLA allele after epitope prediction of a specific antigen/peptide and weighted the data to HLA allele positive rate. Then summed over these data to get the average epitope number (AEN) of a specific antigen/peptide in the population (S3–S6 Tables).

**Fig 2:**
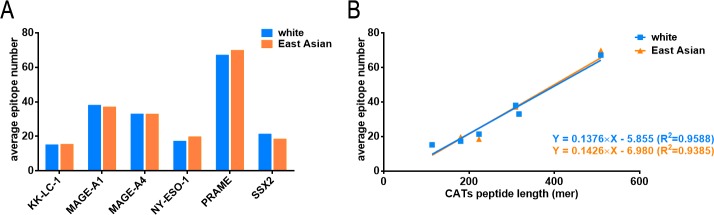
SB epitope prediction of CTAs in white and East Asian populations. A. Average epitope numbers of 6 CTAs. B. Linear analysis between average epitope numbers and antigen lengths. The linear equations and R square values are given in the figure.

In the part of epitope prediction of the 6 CTAs, both white and East Asian populations have the similar AENs after HLA allele positive rate weighting (Fig 2A). Moreover, the AENs of both populations have an excellent linear correlation with the peptide length of the antigens (Fig 2B).

**Fig 3:**
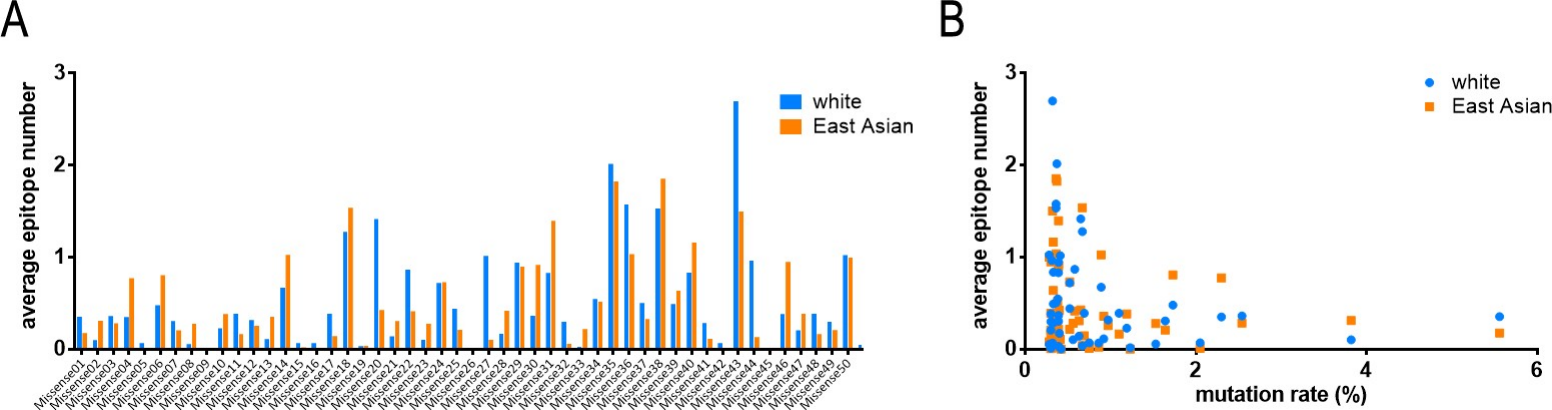
SB epitope prediction of single amino acid mutations in white and East Asian populations. A. Average epitope numbers of 50 missense mutations. B. Correlation analysis between average epitope numbers and mutation rates.

**Fig 4:**
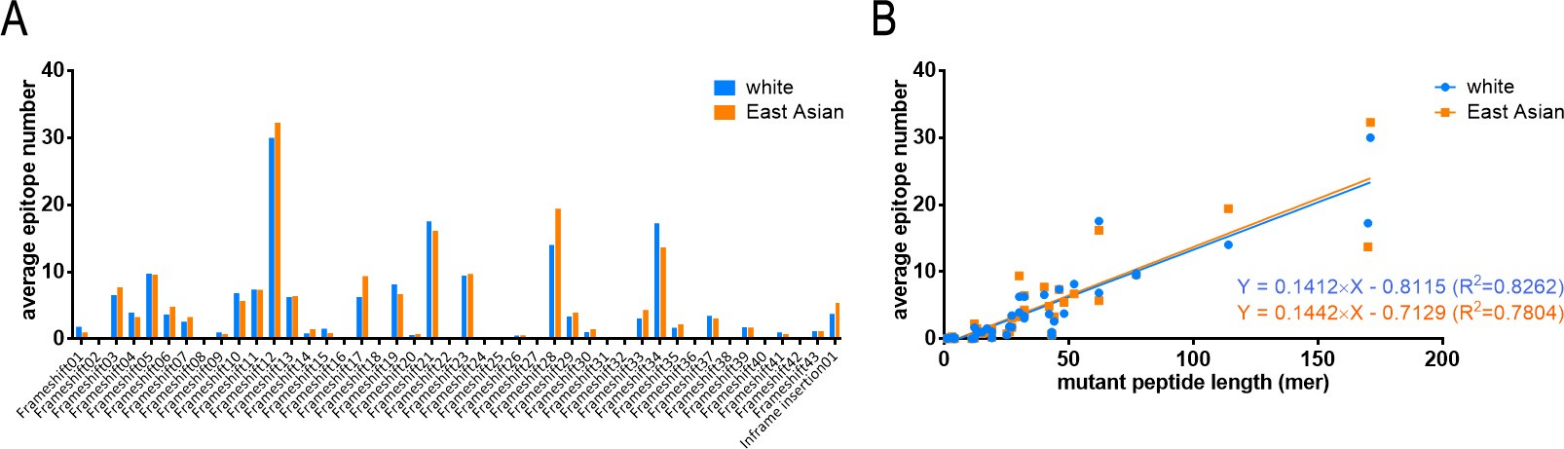
SB epitope prediction of frame related mutations in white and East Asian populations. A. Average epitope numbers of 43 frameshift mutations and 1 inframe insertion mutation. B. Linear analysis between average epitope numbers and mutant peptide lengths. The linear equations and R square values are given in the figure.

**Fig 5:**
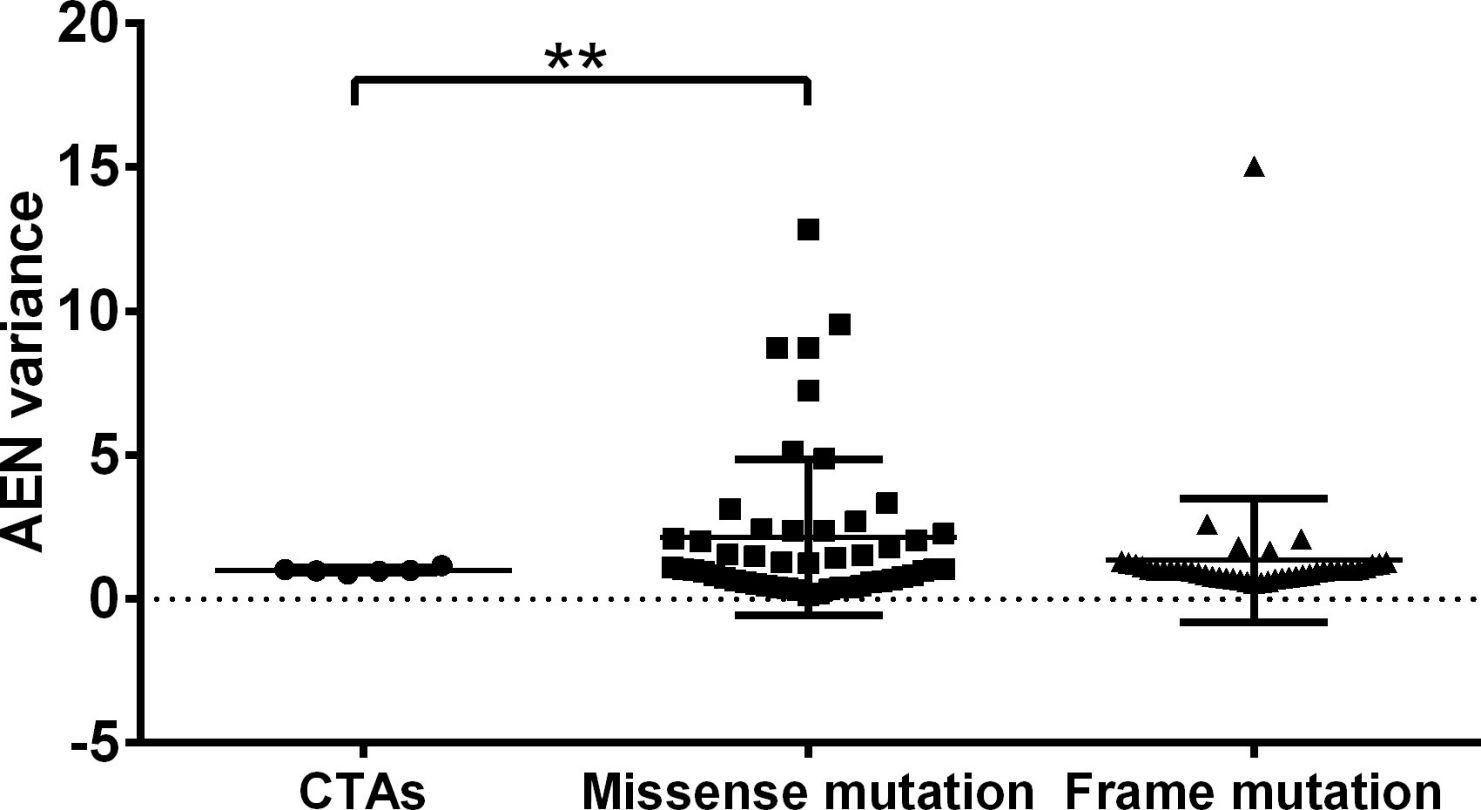
Comparison of average epitope number fold variances between white and East Asian populations in CTAs, neoantigens of missense mutations and frameshift/inframe insertion mutation. Mutations with AEN value 0 in both populations were set to have AEN variance values of 1. Frame mutations in the diagram includes frameshift and inframe insertion mutations. Unpaired t-test was used, **p<0.*01*.

For the epitope prediction of tumor neoantigens, the AENs of missense mutations were quite different between the two populations (Fig 3A). For example, Missense20 (GTF2I_L424H), Missense27 (TP53_G245S), Missense44 (PPP2R1A_P179R). Diagram indicates that, in both populations, the three top missense mutations including BRAF_V600E, IDH1_R132H, PIK3CA_E545K (all over the rate of 2.5%) show low AENs, while the three highest AENs are all uncommon mutations including TP53_V157F (0.32%), ERBB2_S310F (0.37%), PIK3CA_H1047L (0.36%) (Fig 3B). The AENs of frameshift/inframe insertion mutations are similar between two populations (Fig 4A). In addition, AENs of different frameshift/inframe insertion mutations within one population are correlated with the lengths of mutated amino acids (Fig 4B), although the linear relation is not as obvious as in CTAs. Comparison of AENs fold difference of the two populations in three kinds of antigens (non-mutated and mutated) showed that there is a significant difference between CTAs and missense mutations but not the other two pairwise comparisons (Fig 5). These indicated that frameshift/inframe insertion mutations show similar characteristics of both CTAs and missense mutation.

## Discussion

Specific immunotherapy of both adoptive T cell therapy and cancer vaccine involves in CD8+ CTL induction *in vitro* or *in vivo* by antigen stimulation, which is one of the key factors affecting immunotherapy effects. For the selection of optimal peptide pool (with high affinity), it needs to predict tumor antigen epitope accurately. NetMHC is one of the most commonly used epitope prediction algorithms developed by DTU Bioinformatics. The accuracy of the top predicted peptide is about 34% in version 4.0 of NetMHC [17]. There are only 81 HLA allele options in this version, while NetMHCpan could cover most of the known HLA alleles. Therefore, in this study we chose NetMHCpan4.0 as the unified algorithm.

The HLA allele frequency varies widely among different races. This would lead to the same tumor antigen having different CD8+ T cellular immunogenicity in different populations. In this study, we predicted and compared the specific epitopes of CTAs and neoantigens in white and East Asian population, respectively. The results showed that there was a good linear relationship between AENs and the peptide lengths of the CTAs. Meanwhile, the AEN differences of CTAs were small between the two populations. Frameshift/inframe insertion mutation antigens showed the similar trends but the linearity was worse. Although with the same mutated length, the AENs of missense mutations were very different between each other in each population. Moreover, the AENs of many missense mutations vary greatly in two populations. It is worth noting that the single point mutations with the highest incidence have the lowest AEN while the mutations with the highest AEN scores are with low mutation incidence. This may be explained by the immune selective pressure and evolution. Hot mutations are often from cancer driver genes and need to be low immunogenic to avoid the host immunosurveillance. Therefore, the somatic mutation with high immunogenicity must be scarce at the population level. These mutations are often passenger mutations and are with high individualization. The neoantigen related immunotherapy researches so far indicate that the vast majority of antigen specific CD8+ T cells are targeting passenger mutations.

The effective tumor specific CD8+ T cell stimulation involves factors of HLA allele, tumor antigen epitope and TCR repertoire. These factors vary greatly among cancer patients. In terms of tumor antigen, mutation neoantigens have greater individual differences and less potential epitopes than TAA. Therefore, the valuable mutation neoantigens will be with high individualization, which reduces the versatility of neoantigen vaccine [7, 18]. In order to reduce costs and improve efficiency in clinical immunotherapy, one viable solution, we could design and produce common tumor antigen peptides or specific TCR-T cells in advance [19]. The result of this study shows that there are few neoantigens fulfilling the requirement to have both high immunogenicity and high incidence at the same time. However, CTAs could make up for the shortage of neoantigens [20]. CTAs have no mutation that reduced the personalization differences in population. Meanwhile, the long antigen sequence of CTAs ensures that they would always contain epitopes being presented by different HLA alleles. It is possible that the form of full-length CTAs-mRNA vaccine can be applied in different populations. This will greatly reduce the preparation time and cost in tumor immunotherapy [21].

## Conclusion

This study predicted the epitopes of CTAs and neoantigens from the top mutations that were presented by the common HLA class I molecules in both white and East Asian populations. Epitopes analysis showed the immunogenicity of CTAs and neoantigens and their potential values in cancer vaccine design.

## Supporting information

S1 Table Mutation peptide sequences. 95 mutations selected for neoantigen epitope prediction.
(XLSX)

S2 Table HLA allele analysis of white and East Asian. Population samples selected in HLA allele analysis, HLA allele frequencies and positive rates in white and East Asian populations.
(XLSX)

S3 Table CTAs epitope prediction. SB epitope prediction of CTAs and average epitope number (AEN) analysis in white and East Asian populations.
(XLSX)

S4 Table Missense mutations epitope prediction. SB epitope prediction of missense mutations and AEN analysis in white and East Asian populations.
(XLSX)

S5 Table Frameshift mutations epitope prediction. SB epitope prediction of frameshift mutations and AEN analysis in white and East Asian populations.
(XLSX)

S6 Table Inframe insertion mutations epitope prediction. SB epitope prediction of inframe insertion mutation and AEN analysis in white and East Asian populations.
(XLSX)
